# Notch1 regulates the JNK signaling pathway and increases apoptosis in hepatocellular carcinoma

**DOI:** 10.18632/oncotarget.17434

**Published:** 2017-04-26

**Authors:** Chengxu Sui, Chengjun Zhuang, Deguang Sun, Li Yang, Liang Zhang, Lei Song

**Affiliations:** ^1^ Department of Interventional Therapy, The Second Affiliated Hospital of Dalian Medical University, Dalian, 116027, Liaoning, China; ^2^ Department of Intensive Care Unit, The Second Affiliated Hospital of Dalian Medical University, Dalian, 116027, Liaoning, China; ^3^ Department of Hepatobiliary Surgery, The Second Affiliated Hospital of Dalian Medical University, Dalian, 116027, Liaoning, China

**Keywords:** Notch1, JNK, HCC

## Abstract

Notch1-induced pathways are involved in cell growth, apoptosis, motility, and invasion in many cancers. In the present study, the expression of Notch1 and NICD1 was detected in hepatocellular carcinoma (HCC) tissues using in-vitro assays. And then, we explored cell biology and signaling pathways using Notch1 siRNA or plasmids. Here, the expression of Notch1 and NICD1 was significantly decreased in HCC tissues. In-vitro, Notch1 plasmids inhibited cell proliferation, migration and invasion, but enhanced apoptosis of HepG2 and Hep3B cells. Conversely, si-Notch1 enhanced cell proliferation, migration and invasion, but inhibited apoptosis of HepG2 and Hep3B cells. Mechanically, Notch1 decreased the expression of cyclin D1, MMP-9 and Bcl-2, but increased the expression of p-JNK, Bax and cleaved caspase 3 in HepG2 and Hep3B cells. Besides, si-JNK or JNK inhibitor SP600125 affected the activation of Notch1 signaling pathway, and prevents cell apoptosis. In conclusion, Notch1 regulates the JNK signaling pathway and increases apoptosis in HCC. Because patients with HCC have a poor prognosis, Notch1 pathway may provide a novel treatment strategy.

## INTRODUCTION

HCC is reported as the most common one in digestive cancers in the worldwide. Every year, about a half of patients and dead occurred in some countries, especially in China [1, 2]. Despite cancer tissues of several patients underwent operative resection, these patients still suffered from a poor prognosis [3]. Notably, some HCC patients with advanced stage have no chances for operation [4], and their overall survival period is less than one year [5]. It has been reported that recurrence and metastasis accounts for the high mortality of HCC patients [6]. To date some anti-cancer therapy and various surgical interventions have been introduced, while the overall effects are not satisfying. Thus, it is essential to explore the molecular mechanisms of HCC.

Notch signaling plays an important role in the tumor biology, including cell differentiation, proliferation, apoptosis, migration and invasion [7–10]. Increasing reports demonstrated that deregulation of Notch1 signaling can be observed in diverse tumor types, such as cervical cancer. [11–13]. The activation of Notch signaling in cervical cancer plays an important role in regulating tumor cell proliferation and apoptosis, thus, targeting of Notch signaling may offer us a useful and effective therapy strategy for cervical cancer patients [12]. Generally speaking, Notch pathway is much more complex, and exerts different effects on different stages of tumor progression, including an increase in early stage of tumors and a decrease in the late stage of tumors [13]. However, the molecular mechanisms of Notch pathway are not completely known till now.

In this study, we investigated the effects of Notch1 pathway on cell proliferation, apoptosis, and biological behaviors of HCC cells. Our results showed that suppression of expression of Notch1 can enhance the proliferation, and induce cell apoptosis and invasiveness of HCC cells, suggesting that Notch1 is a potential and useful target for the clinical treatment of patients with HCC.

## RESULTS

### Expression of Notch1 and NICD1 decreases in HCC tissues

**Figure 1:**
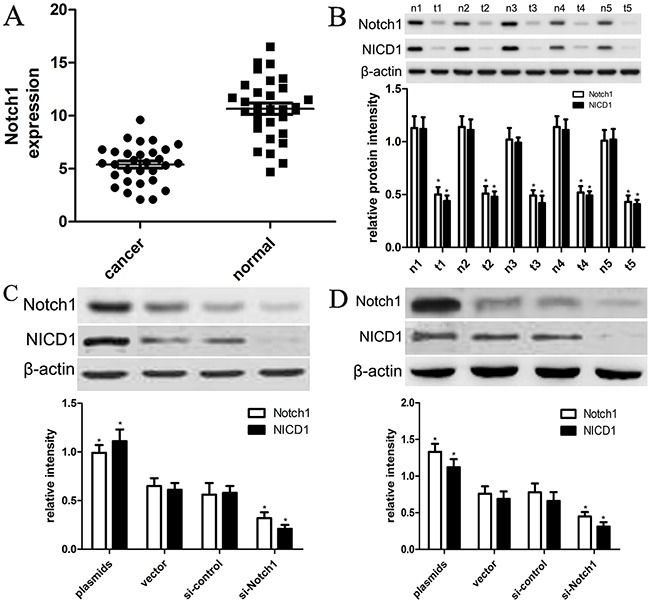
The expression of Notch1 and NICD1 in liver cancer tissues and cells (**A**) Low expression of Notch1 mRNA in tumor tissues than in normal tissues (*p<0.001). (**B**) 5 representative tissues were subjected to the western blot. T=tumor tissues, N=normal tissues (*p<0.001). (**C**) the plasmids and siRNA were used to establish HepG2 and Hep3B cell lines that expressed lower and higher levels of Notch1 and NICD1, respectively (*p<0.001). The vector and control siRNA-transfected cell line was designated as the control group. (**D**) The plasmids and siRNA were used to establish Hep3B cell lines that expressed lower and higher levels of Notch1 and NICD1, respectively (*p<0.001). The vector and control siRNA-transfected cell line was designated as the control group. * denotes significance at p<0.01 relative to control by student t-test.

To figure out expression of Notch1 and NICD1 in HCC cancer tissues and normal tissues in the present study, we carried out qRT-PCR and western blot analysis by extracting their total mRNAs and proteins. In this study, 30 cases of tissue samples were collected and detected using Notch1 primers or anti-Notch1 antibody. We found that the expression level of Notch1 mRNA is 10.3±3.1 in normal tissues, while the expression level of Notch1 mRNA is 5.1±2.1 (Figure 1A); their differences are statistically significant (p<0.001). Furthermore, western blot analysis demonstrated that expression of Notch1 protein was also significantly decreased in tumor tissues compared with that in normal liver tissues (p<0.001) (Figure 1B). In addition, western blot also identified that expression of NICD1 protein was significantly decreased in tumor tissues compared with that in normal liver tissues (p<0.001) (Figure 1B). These findings suggested that Notch1 were involved into the development of HCC.

### Expression of Notch1 is altered by in-vitro plasmids or siRNAs in HepG2 and Hep3B cells

To elucidate the effects of expression of Notch1 on biological behaviors of HCC cell lines, we designed Notch1 siRNAs (si-Notch1) and transfected si-Notch1 into HepG2 and Hep3B cells to interfere with expression of endogenous Notch1. Meanwhile, we transfected control siRNA into HepG2 and Hep3B cells. Afterwards, the expression of Notch1 was detected using western blot analysis. As shown in Figure 1C, 1D, si-Notch1 effectively and successfully inhibited expression of Notch1 and NICD1 in HepG2 and Hep3B cells compared with si-control (p<0.001). Besides, we also used Notch1 plasmids and vector control to establish overexpression of Notch1 in HepG2 and Hep3B cells. The cell lines transfected with vector control were indicated as control. As shown in Figure 1C-1D, we found that Notch1 plasmids effectively promoted expression of Notch1 and NICD1 in HepG2 and Hep3B cells compared with vector control (p<0.001).

### Notch1 regulates cell proliferation of HepG2 and Hep3B cells

**Figure 2:**
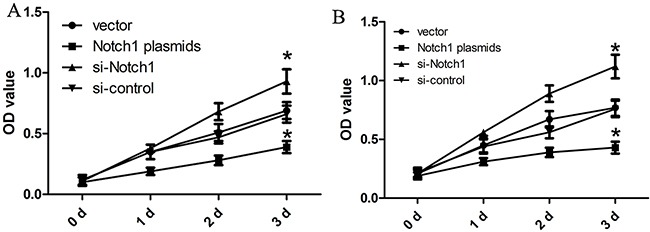
Notch1 affects the proliferation of liver cancer cells The HepG2 (**A**) and Hep3B (**B**) cells that were transfected with Notch1 plasmids or siRNA displayed significant growth changes compared with the cells that were transfected with the control after 24 h (*p<0.001). * denotes significance at p<0.01 relative to control by student t-test.

In order to explore biological role of Notch1 in the progression of liver cancer, we carried out a CCK8 assay when HepG2 and Hep3B cells were transfected with Notch1 siRNA or control siRNA. As shown in Figure 2, HepG2 and Hep3B cells transfected with si-Notch1 showed a significant growth improvement compared with those transfected with si-control after 24 h (both p<0.001). However, HepG2 and Hep3B cells transfected with Notch1 plasmids showed a significant growth inhibition as compared with vector control after 24 h (both p<0.001).

### Notch1 facilitates HepG2 and Hep3B cell apoptosis

**Figure 3:**
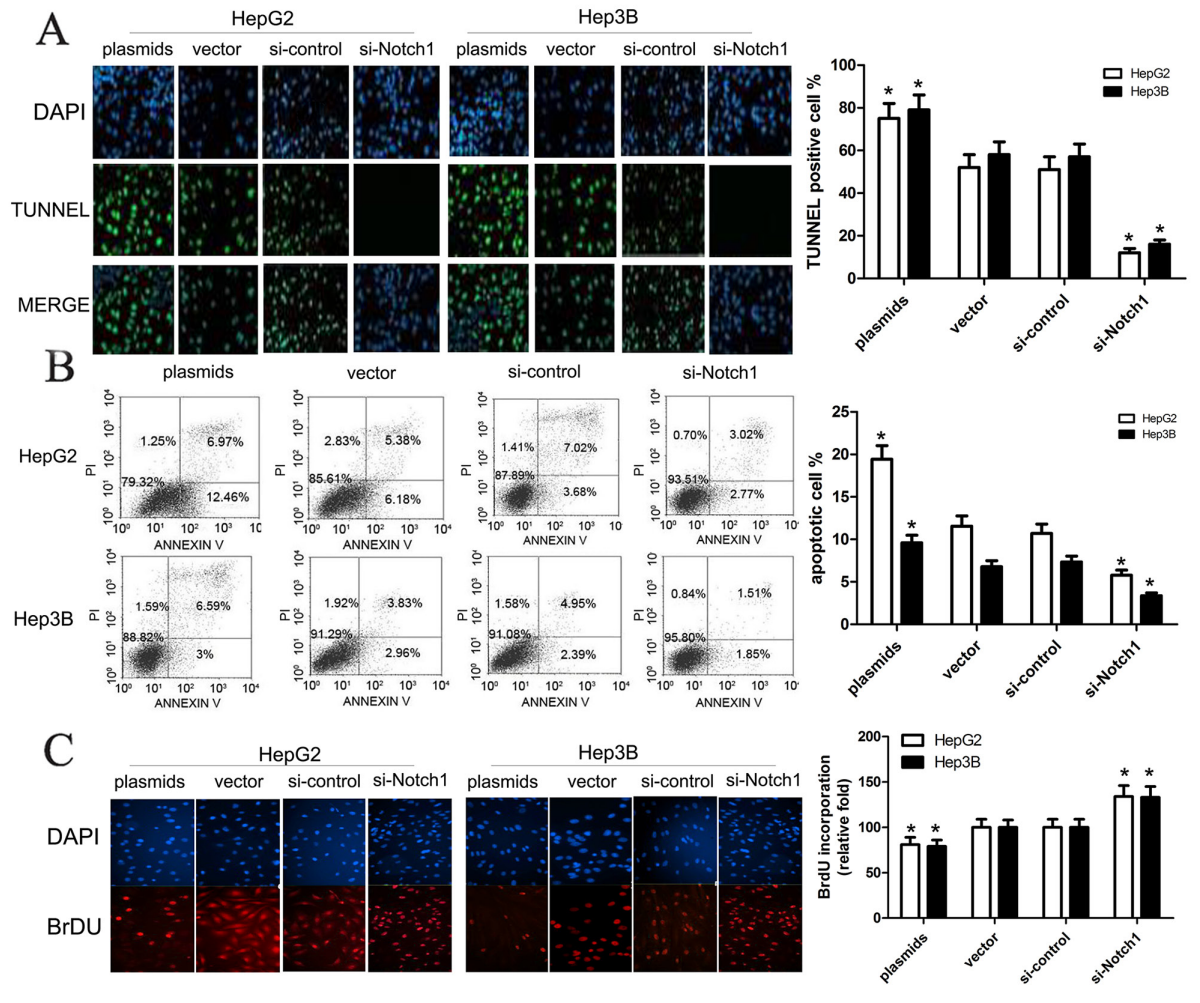
Notch1 affects apoptosis of HepG2 and Hep3B cells (**A**) Apoptotic effects of plasmids and si-Notch1 on HepG2 and Hep3B cells were determined by TUNEL assay (green channel) at 90 minutes after treatment. DAPI (blue channel) is used to locate the nuclei of the cells. (*p<0.001). (**B**) HepG2 and Hep3B cells were transfected with Notch1 plasmids, Notch1 siRNA or the relative controls as indicated for 48 h, and then cells were stained with Annexin V-FITC/PI, and analyzed by flow cytometry as described in methods. The statistic data were presented as mean ± SEM from three independent experiments. Representative images for cell apoptosis stained with Annexin V-FITC/PI. (**C**) Representative micrographs (upper) and quantification (lower) of BrdU incorporation by different-treated cells. * denotes significance at p<0.01 relative to control by student t-test.

**Figure 4:**
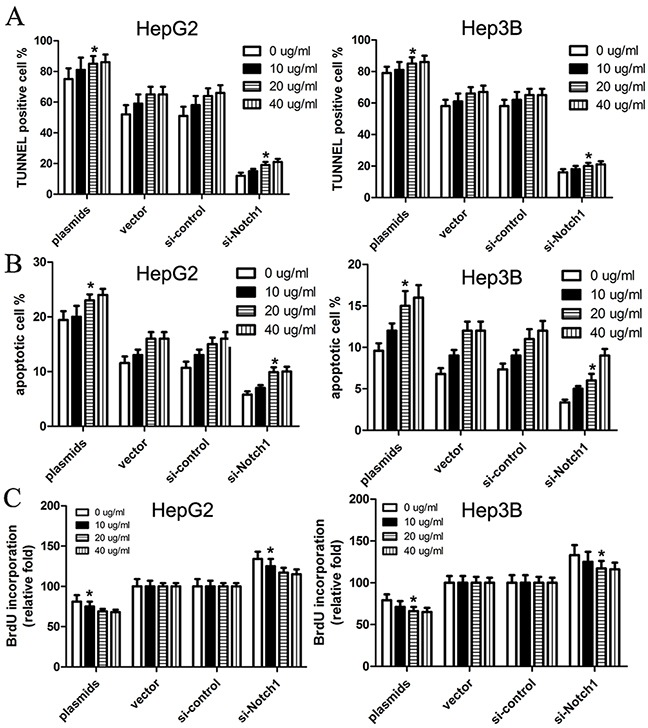
Cisplatin facilitates Notch1-induced cell apoptosis of HepG2 and Hep3B cells We treated cells with apoptosis inducing agent cisplatin (0, 10, 20, 40μg/ml), and then cells were subjected to TUNEL assay (**A**), apoptosis assay (**B**) and BrdU incorporation assay (**C**). The statistic data were presented as mean ± SEM from three independent experiments. * denotes significance at p<0.01 relative to control by student t-test.

With the results above, we used TUNNEL and flow cytometry to conduct cell apoptosis assay. Here, HepG2 and Hep3B cells were transfected with Notch1 siRNA, Notch1 plasmids, or their corresponding controls. TUNNEL assay revealed that TUNNEL positive cell rate in cells with Notch1 plasmids was obviously higher than control (p<0.001), while TUNNEL positive cell rate in cells with Notch1 siRNA was significantly lower that control (p<0.001) (Figure 3A). Consistent with TUNNEL, flow cytometry revealed that the apoptotic rate of HepG2 and Hep3B cells transfected with Notch1 plasmids was 19.4% and 9.3%, respectively; while the apoptotic rate of HepG2 and Hep3B cells transfected with vector control was 11.6% and 6.8%, respectively. In addition, the apoptotic rate of HepG2 and Hep3B cells transfected with si-Notch1 was 5.8% and 3.4%, respectively; while the apoptotic rate of HepG2 and Hep3B cells transfected with control was 10.7% and 7.3%, respectively (Figure 3B). As expected, BrdU incorporation assay revealed that Notch1 reduced BrdU incorporation of HepG2 and Hep3B cells (Figure 3C). After optimization of transfection, we challenged cells with apoptosis inducing agent cisplatin to see whether Notch1 overexpressing cells are more susceptible to apoptosis than Notch1 knockdown cells. The results revealed that the cell apoptosis was obviously enhanced in all four groups following increased cisplatin concentrations, and difference of cell apoptosis was not statistically significant between 20 μg/ml and 40 μg/ml cisplatin (Figure 4). However, Based on incremental difference in Notch1 plasmids and si-Notch group, we found that Notch1 overexpressing cells were not more susceptible to apoptosis than Notch1 knockdown cells (p>0.05).

**Figure 5:**
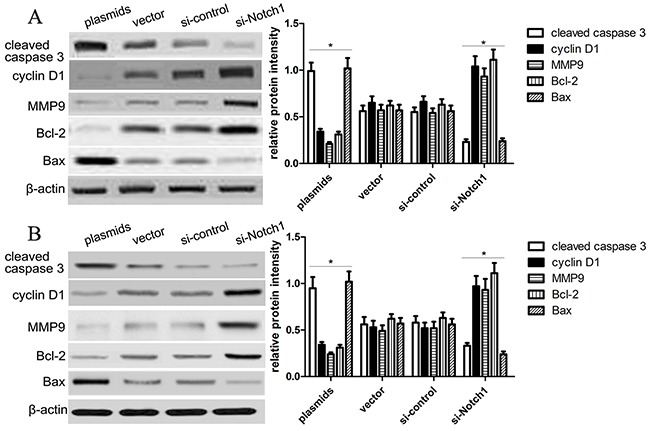
Notch1 affects proliferation and apoptosis-related gene expression (**A**) Western blot analysis showed the expression of cleaved caspase-3, cyclin D1, MMP9, Bcl-2 and Bax of HepG2 cells was changed in the plasmids and si-Notch1 group than those in the control groups (*p<0.001). The statistic data were presented as mean ± SEM from three independent experiments. (**B**) Western blot analysis showed the expression of cleaved caspase-3, cyclin D1, MMP9, Bcl-2 and Bax of Hep3B cells was changed in the plasmids and si-Notch1 group than those in the control groups (*p<0.001). * denotes significance at p<0.01 relative to control by student t-test.

Then, to further confirm the impact of Notch1 pathway on HepG2 and Hep3B cell growth and apoptosis, the expression of proliferation and apoptosis-related biomarkers, including Bcl-2, cyclin D1, caspase-3 and Bax, was detected using western blotting analysis. Our findings revealed that Notch1 increased the expression of cleaved-caspase-3 and Bax, but decreased the expression of cyclin D1 and Bcl-2 in HepG2 and Hep3B cells (Figure 5). Besides, we also observed that the expression of MMP9 was significantly decreased in HepG2 and Hep3B cells transfected with Notch1 plasmids, but increased in HepG2 and Hep3B cells transfected with si-Notch1 group, suggesting that Notch1 might affect the migration and invasion of HepG2 and Hep3B cells.

### Notch1 affects migration and invasion of HepG2 and Hep3B cells

**Figure 6:**
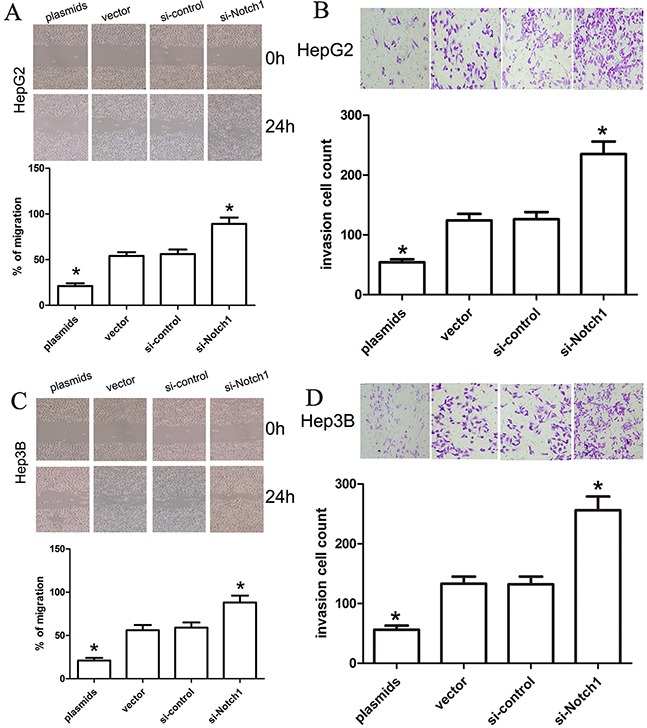
Notch1 affects the migration and invasion ability of liver cancer cells A change in HepG2 and Hep3B cell migration (**A, C**) and invasion (**B, D**) was observed in the Notch1 plasmids or siRNA-transfected liver cancer cells (*p<0.001). * denotes significance at p<0.01 relative to control by student t-test.

To investigate the effect of Notch1 on migration and invasion of HepG2 and Hep3B cell lines, we carried out in-vitro migration and invasion assays using HepG2 and Hep3B cells transfected with si-Notch1, Notch1 plasmids and their corresponding controls. In the present study, cells that migrated across the dash line or invade through Matrigel membrane were counted, and we found that Notch1 plasmids obviously decreased cell migration and invasion in HepG2 and Hep3B cells compared with vector control (Figure 6), while si-Notch1 increased cell migration and invasion in HepG2 and Hep3B cells compared with si-control (Figure 6), which indeed demonstrated that Notch1 affects migration and invasion of HepG2 and Hep3B cells.

### Notch1 activates JNK phosphorylation in HepG2 and Hep3B cells

**Figure 7:**
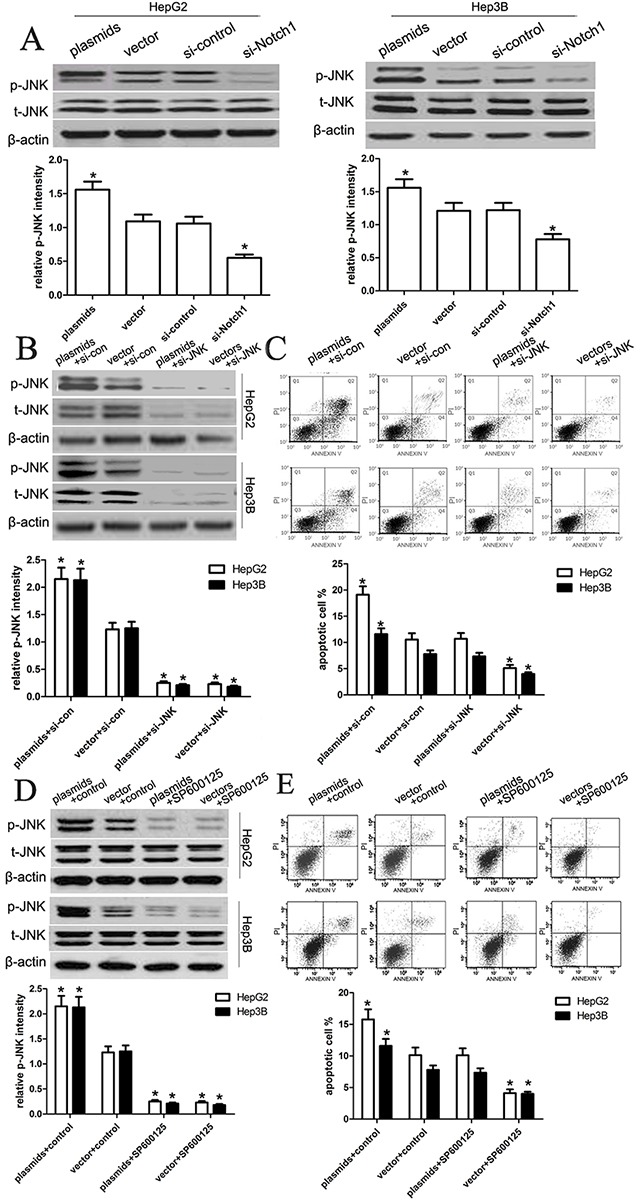
Notch1 regulates JNK phosphorylation in liver cancer cells (**A**) After transfection of the HepG2 and Hep3B cell lines, we tested the changes of total JNK levels and JNK phosphorylation. The knockdown of Notch1 significantly reduced the phosphorylation of JNK in both HepG2 and Hep3B cell lines, which is consistent with the general understanding that JNK is activated in transformed cells. However, the overexpression of Notch1 obviously enhanced the phosphorylation of JNK in both HepG2 and Hep3B cell lines (*p<0.001). (**B, C**) We transfected the si-JNK or si-control into HepG2 and Hep3B cells with Notch1 plasmids, and then the expression of p-JNK and t-JNK, and cell apoptosis were tested. The statistic data were presented as mean ± SEM from three independent experiments. * denotes significance at p<0.01 relative to control by student t-test. (**D, E**) We treated HepG2 and Hep3B cells with JNK inhibitor SP600125, and then the expression of p-JNK and t-JNK, and cell apoptosis were tested. The statistic data were presented as mean ± SEM from three independent experiments. * denotes significance at p<0.01 relative to control by student t-test.

To elucidate the molecular mechanisms underlying Notch1-induced apoptosis, we further analyzed the expression and phosphorylation level of JNK in HepG2 and Hep3B using western blot analysis. We assumed that JNK pathway might be inducibly activated to mediate cell apoptosis. Following the transfection of HepG2 and Hep3B cell, we evaluated changes in the expression and phosphorylation level of JNK (Figure 7A). As expected, Notch1 plasmids significantly activated the phosphorylation of JNK in both cell lines as compared with vector control (p<0.001), while si-Notch1 significantly inhibited the phosphorylation of JNK in HepG2 and Hep3B cell lines (p<0.001). To further identify the Notch1-JNK pathway, we inhibited the expression and activation of JNK using si-JNK or JNK inhibitor SP600125, and found that si-JNK or JNK inhibitor SP600125 significantly inhibited the expression and activation of p-JNK in HepG2 and Hep3B cells with Notch-1 plasmids (p<0.001) (Figure 7B, 7D). Next, we examined the amount of apoptosis using flow cytometry, and found that si-JNK or JNK inhibitor could prevent cell apoptosis induced by Notch1 plasmids (Figure 7C, 7E). These results indicated that Notch1 activates JNK phosphorylation to induce apoptosis in HepG2 and Hep3B cells.

## DISCUSSION

To date, a number of reports have demonstrated that the expression of Notch family is aberrant, and often observed to be activated in some human malignancies such as cervical cancer, and pancreas cancer [14–16]. Moreover, the expression of Notch-1 and its ligand Jagged-1 is reported to be related to poor prognosis in breast and prostate cancer [17–21]. Notch pathway is much more complex, and exerts different effects on different stages of tumor progression, including an increase in early stage of tumors and a decrease in the late stage of tumors. Recent studies demonstrated that Notch1 is involved in tumorigenesis, and regulated some tumor biology processes, including the cell proliferation, apoptosis, migration, and invasion [22–25], indicating that Notch1 plays an important role in early detection of cancer, and acts as a therapeutic target and a useful biomarker. However, the molecular mechanisms of Notch pathway have not been completely known till now.

In the present study, we identified that the expression of Notch1 protein went lower in HCC cancer tissues compared with that in non-tumor tissues. Based on these findings, our study aims to elucidate the molecular mechanisms of Notch1-induced apoptotic signaling pathways that regulate biological behaviors of HCC cells. We established Notch1 plasmids and siRNA to enhance or suppress expression of Notch1, and identified the regulation role of Notch1 in cell proliferation, migration and invasion. Because the Notch1-JNK pathway-induced apoptosis has been reported widely, we further explored the molecular mechanisms of Notch1 in HCC tumorigenesis.

JNKs were reported as the stress-activated protein kinases in the mouse liver administrated with cycloheximide, and activation of JNKs induces inflammation and apoptosis [26]. JNKs have a close relationship with c-Jun, a kind of phosphorylation-activated transcription factor. It has been demonstrated that JNKs consist of three encoded genes in human, such as Jnk1, Jnk2 and Jnk3, among which, Jnk1 and Jnk2 are extensively found in human tissues while Jnk3 is specifically found in the brain, heart and testis tissues. JNKs can be triggered by a variety of stressors, involving UV irradiation and oxidative stress, leading to cell apoptosis or inhibition of cell growth [27]. When activated, JNKs protein shuttles from cytoplasm to nucleus where it induces activation of the transcription factor c-Jun [28]. c-Jun has been reported to modulate expression of apoptosis related genes, including Bcl2-associated X protein and B-cell lymphoma 2 [29,30]. JNK can phosphorylates amino acid residues 63 and 73 located at N-terminal of c-Jun proteins, and then activated its transcriptional activity. Many studies have reported the close relationship between JNK and c-Jun in tumor biology.

In this study, we assumed that JNK pathway might be inducibly activated to mediate cell apoptosis. Following the transfection of HepG2 and Hep3B cell, we evaluated changes in the expression and phosphorylation level of JNK. As expected, Notch1 plasmids significantly activated the phosphorylation of JNK in both cell lines as compared with vector control, while si-Notch1 significantly inhibited the phosphorylation of JNK in HepG2 and Hep3B cell lines, which indicated that Notch1 activates JNK phosphorylation in HepG2 and Hep3B cells, by which Notch1 affected cell apoptosis. This finding suggests that JNK/c-jun pathway is involved in regulation of Notch1-induced apoptosis. Furthermore, to ascertain the mechanisms of apoptosis induced by Notch1, we detected the expression level of caspase-3, and cyclin D1 proteins, and demonstrated that Notch1 plasmids increased the expression of cleaved-caspase-3, but decreased the expression of cyclin D1 in HepG2 and Hep3B cells. Besides, we also observed that the expression of MMP9 was significantly decreased in HepG2 and Hep3B cells transfected with Notch1 plasmids, but increased in HepG2 and Hep3B cells transfected with si-Notch1 group, suggesting that Notch1 indeed affects the migration and invasion of HepG2 and Hep3B cells.

It should be noted that recent studies showed Notch1 as a tumorigenic factor in HCC. Villanueva A et al reported that Notch expression is activated in human HCC tissues, and Notch expression facilitates development of liver tumors in the mice model [31]. Giovannini C et al reported that targeting Notch1 can be used for clinical therapy of hepatocellular carcinoma [32]. Han B et al also suggested that Notch1 siRNA combined with IL-24 can sensitize cell apoptosis and reduce HepG2 cell invasion and migration [33]. Together with our study, we assumed that Notch1 has different roles in different stage of HCC development, and Notch1 expression is regulated by some unknown factors in HCC development, leading to diverse biological functions.

In conclusion, we determined that Notch1 regulates the JNK signaling pathway and increases apoptosis in hepatocellular carcinoma. Because patients with HCC have a poor prognosis, Notch1 pathway may provide a novel treatment strategy.

## MATERIALS AND METHODS

### Ethical statement

All procedures performed in studies involving human participants were in accordance with the ethical standards of the institutional and/or national research committee and with the 1964 Helsinki declaration and its later amendments or comparable ethical standards.

### Patients and specimen selection

Paraffin-embedded pathological specimens were obtained from the archives of the Department of Pathology of The Second Affiliated Hospital of Dalian Medical University (People's Republic of China) between January 2013 and January 2015. In all, 30 samples of liver cancer were obtained. In addition, 30 samples of normal liver tissues that were obtained from patients who underwent surgery were included in this study. Approval for the current project was obtained from the local ethics committee, together with written informed consent from each patient. The patients were aged 28–70 years (median, 46 years). The consent procedure and study protocol were approved by the Medical Institutional Ethical Committee of The Second Affiliated Hospital of Dalian Medical University.

### Cell culture and reagent

The liver cancer cell lines HepG2 and Hep3B used in the present study were obtained from the Chinese Academy of Sciences (Shanghai, China). All cell lines were cultured in RPMI-1640 supplemented with 10% fetal bovine serum (FBS), penicillin G (100 U/mL), and streptomycin (100 g/ml) and were maintained in monolayer culture at 37°C in humidified air with 5% CO2. The JNK inhibitor SP600125 was obtained from Santa Cruz (5142-23-4).

### SiRNA transfection

Small interfering RNA that targeted Notch1-RNA (Notch1-siRNA) and a scrambled negative control (Scrambled-SiRNA) were generously provided by Life Technologies. Cells were transfected with either 50 nmol Notch1-siRNA or Scrambled-SiRNA using Lipofectamine 2000 transfection reagent according to the manufacturer's protocol (Life Technologies). The sequences were as follows: Notch1, Forward 5′-GTCTCCATTGCTAGCCAC-3′ and Reverse 5′-ATGCAGCTGCAGGTCTTAAGAG-3′; GAPDH, Forward 5′-ACAGGGGAGGTGATAGCATT-3′ and Reverse 5′-GACCAAAAGCCTTCATACATCTC-3′. Notch1-SiRNA: GUCCAGGAAACAACUGCAATT (sense) and UUGCAGUUGUUUCCUGGA CTT (antisense). Scrambled-SiRNA: UUCUCCGAACGUGUCACGUTT (sense) and ACGUGA CACGUUCGGAGAATT (antisense).

### Plasmid transfection

Cells (2×105 per well) were seeded into 60 mm plates 24 hours prior to plasmid transfection. Cells were then transfected with the empty vector (pCMV6-Entry, Origene, Rockville, MD), or the human cDNA ORF clone of Notch1 (pCMV6-Notch1) using MegaTran 1.0 (Origene) for 6 hours in Opti-MEM® I Reduced Serum Medium (Invitrogen). Cells were harvested at the indicated time points.

### Quantitative real-time PCR assay

Total RNA was isolated from tissues using TRIZOL reagent (Invitrogen) or cells using Trizol plus kit (TaKaRa, Japan) according to the manufacturer's protocol. RNA was reverse transcribed using SuperScript First Strand cDNA System (Invitrogen) according to the manufacturer's instructions. The PCR amplification were performed for 40 cycles of 94°C for 30 s, 60°C for 30 s, and 72°C for 30 s, on a Applied Biosystems 7900HT (Applied Biosystems) with 1.0 μl of cDNA and SYBR Green Real-time PCR Master Mix (Takara). Data was collected and analyzed by SDS2.3 Software (Applied Biosystems). The expression level of each sample was internally normalized against that of the GAPDH. The relative quantitative value was expressed by the 2-ΔΔCt method. Each experiment was performed in triplicates.

### Western blot analysis

Tissues or cells were rinsed with cold PBS and harvested in lysis buffer. Then, the extractions were obtained and then centrifuged at 14,000 rpm for 30 min. Twenty-five micrograms of protein was loaded per lane and separated by 10% SDS-PAGE, then transferred to nitrocellulose membranes and blocked overnight in Trisbuffered saline containing 0.1% Tween and 5% skim milk. Then, the membrane was incubated with primary antibodiesat 4°C and subsequently incubated with a secondary antibody for 2 h at room temperature. Primary antibodies against phosphorylated JNK, t-JNK, and NICD (Cell Signaling Technology, Beverly, MA, USA), as well as cyclin D1, Bcl-2, Bax, MMP9 and β-actin (Santa Cruz Biotechnology, Santa Cruz, CA, USA) were diluted 1:1000. Horseradish peroxidase-conjugated secondary antibodies (Santa Cruz Biotechnology, Santa Cruz, CA, USA) were diluted 1:7000. Finally, the detection of specific proteins was carried out using an ECL western blotting kit (Amersham Biosciences, Piscataway, New Jersey, USA) according to the recommended procedure.

### CCK8 assay

The transfected liver cancer cells were seeded into 96-well plates (Corning Incorporated, Corning, New York, NY, USA) at a density of 3,000 and 5,000 cells per well for HepG2 and Hep3B cells, respectively, where each well contained medium supplemented with 10% FBS. The cultures were stained using a cell counting kit-8 (CCK-8; Beyotime Institute of Biotechnology, Haimen, Jiangsu, China) at various time points. Briefly, 20 μl of CCK-8 solution was added to each well, and then incubate the solution for 4 h at 37°C. Each solution was then measured by spectrophotometry at 450 nm in a Multiskan Ascent microplate reader (ELx800; Bio-Tek Instruments, Inc., Winooski, VT, USA).

### TUNEL assay

Assessment of cell apoptosis was conducted via Fluorometric TUNEL assay (Promega, Madison, Wisconsin) according to the manufacturer's instructions. The percentage of TUNEL positive cells were calculated by counting fluoresce in positive nucleus and the total number of cells (DAPI labeled nuclei) using Image J software (NIH, Bethesda, MD). At least 8 high power fields (200× magnification) from each glass bottom dish were acquired and analyzed for quantification.

### Apoptosis assay

The cells were collected at 48 h, 72 h and 96 h post transfection respectively. The cells were centrifuged and resuspended in 500 μl of staining solution (containing annexin V fluorescein and propidium iodide in HEPES buffer) (annexin V: FITC apoptosis detection kit; BD PharMingen, San Diego, CA). After incubation at room temperature for 15 min, cells were analyzed by flow cytometry.

### Flow cytometry analysis

Flow cytometry was used to quantitate the percentage of apoptotic cells. For transfection with plasmid DNA, cells were plated in 6-well plates in antibiotic free growth medium at a density of 1 × 106 cells/well. After 24 hours, the transfection was performed using Lipofectamine 2000 according to the manufacturer's instructions.

### Bromodeoxyuridine (BrdU) incorporation assay

Cells (5 × 104) were plated on coverslips. After 24 hours, cells were incubated with BrdU for 1 h and stained with anti-BrdU antibody (Upstate, Billerica, MA) according to the manufacturer's instruction. After washing three times with PBS containing 1% Triton X-100, the cells were treated with anti-mouse TRITC fluorescent conjugated secondary antibodies to visualize anti-BrdU labeled cells. BrdU positive cells were counted under a laser scanning microscope (Axioskop 2 plus; Carl Zeiss Co. Ltd.) in ten random chosen fields from three independent samples. Percentage of BrdU positive cells was then calculated, and the results are presented as the mean ± SD.

### Cell migration and invasion assay

Wound healing assay was used to examine cell migration. Briefly, cells in different groups were cultured to confluence. Wounds of approximately 1 mm width were created using a plastic scriber. Cells were then washed using DPBS, and cultured in serum-free DMEM for 24h. After that, cells were cultured in DMEM with 10% FBS for 36h. Then, cells were fixed and observed under a microscope. Cell invasion were detected by Transwell methods. With regard to the invasion assay, liver cancer cells (2×10^5^) were seeded in the upper chambers in serum-free media with the Matrigel membrane, whereas the lower chambers were loaded with RPMI-1640 supplemented with 10% FBS. After 36 h or 48 h respectively, the cells in the upper chambers that had not migrated were removed by a cotton swab. Cells were fixed with 4% polyoxymethylene. The total number of cancer cells was counted after they were fixed and stained with 4% hematoxylin.

### Statistical analysis

All data in this study are expressed as the mean ± SD of at least three independent experiments. Statistical analysis of differences was performed by one-way analysis of variance (ANOVA) or student t test using SPSS 17.0 software. P value less than 0.05 was considered statistically significant.
